# Post‐Occlusion Application of Skin Protective Creams Does Not Affect Occlusion‐Induced Barrier Changes: A Randomized Controlled Experimental Study

**DOI:** 10.1111/cod.70220

**Published:** 2026-07-15

**Authors:** Michal Gina, Katharina Wichert, Thomas Behrens, Thomas Brüning, Julia Krabbe

**Affiliations:** ^1^ Ruhr University Bochum, Medical Faculty, Institute for Prevention and Occupational Medicine of the German Social Accident Insurance Institute of the Ruhr University Bochum (IPA) Bochum Germany

**Keywords:** barrier disturbance, glove occlusion, irritant contact dermatitis, irritation, non‐invasive measuring methods, occupational contact dermatitis, skin barrier disturbance, skin protective cream, wet work

## Abstract

**Background:**

Glove occlusion increases skin susceptibility to irritants (‘occlusion effect’). Previous experimental data showed that applying protective creams (PCs) before glove occlusion does not prevent this effect and may even aggravate irritant responses.

**Objectives:**

To assess whether the application of PCs after glove occlusion modifies the occlusion effect.

**Methods:**

In a randomized, single‐blind (participants were blinded) study, 68 healthy volunteers were examined over 10 days. Two commercially available PCs (A, B) and one standardized model cream (C) were applied after occlusion for over 4 h to defined forearm sites. On day 8, sites were exposed to 0.5% sodium lauryl sulphate (SLS) for 24 h to evaluate skin irritation susceptibility. Additional conditions included (I) daily application of cream C without occlusion until day 7 or (II) until Day 8, with the final application 10 min before SLS exposure. Skin responses were assessed by TEWL, corneometry, colourimetry and clinical scoring. Wilcoxon signed‐rank tests were used to compare control areas to sides after PC application.

**Results:**

Post‐occlusion application of the PCs did not demonstrate a consistent protective effect. Cream A slightly reduced SLS‐induced TEWL (10.88 g/m^2^/h after application following glove use vs. 12.13 g/m^2^/h without application; adjusted *p* > 0.05), whereas cream B increased TEWL (13.44 vs. 12.42 g/m^2^/h; adjusted *p* > 0.05). Cream C showed no significant effect on TEWL (11.99 vs. 12.38 g/m^2^/h; *p* > 0.05). In contrast, application of cream C shortly before SLS exposure in the absence of occlusion (condition II) significantly reduced TEWL (7.09 vs. 12.38 g/m^2^/h; adjusted *p* < 0.0001) and erythema. However, earlier application (condition I) did not yield a protective effect (11.92 vs. 12.38 g/m^2^/h).

**Conclusions:**

In the majority of study scenarios, application of PCs after glove occlusion did not have a clinically relevant positive effect on the skin responses studied. Protective efficacy probably depends on formulation and timing, underscoring the need for evidence‐based recommendations.

## Graphical abstract

Post‐occlusion application of protective creams did not consistently reduce occlusion‐induced skin barrier impairment after glove use. Protective efficacy probably depends on formulation and timing, with only immediate pre‐irritant application showing a significant reduction in SLS‐induced barrier disruption.

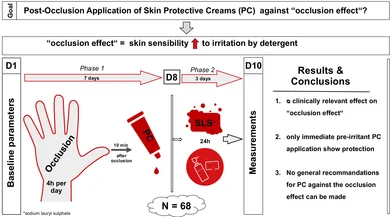

## 1 Introduction

Wet work remains one of the most important occupational risk factors for irritant contact dermatitis (ICD) [1, 2], particularly in professions involving frequent contact with water, detergents, and prolonged use of liquid‐proof gloves [3, 4, 5, 6]. Prolonged occlusion has been shown to induce alterations in skin barrier function, as reflected by increased transepidermal water loss (TEWL) [5, 7], and to enhance hygroscopicity without causing dryness [7]. However, the direct detrimental effects of occlusion alone are generally limited, with significant barrier impairment typically occurring only under conditions of extensive and long‐term occlusion (as reviewed by Tiedermann et al. [8] and Fartasch et al. [9]). In contrast, studies investigating the combined effects of occlusion and exposure to soaps or detergents demonstrate that occlusion can markedly enhance skin barrier damage [10]. This increased susceptibility is characterized by an amplified irritant response to subsequent detergent exposure and is commonly referred to as the ‘occlusion effect’. Clinically, this phenomenon is highly relevant, as it may contribute to the development or exacerbation of ICD under real‐world wet‐work conditions. Reflecting this understanding, the updated German Technical Rules for Hazardous Substances (TRGS 401) have expanded the definition of ‘wet work’ to include combined exposures and alternating cycles of glove use, hand washing, and contact with water or water‐based fluids [11].

To mitigate occupational skin damage, the regular use of skin protective creams (PCs) is widely recommended [12]. Some PC formulations are specifically marketed for application immediately before glove wearing and claim to protect the skin not only from irritants but also from the adverse effects of glove occlusion itself. However, scientific evidence supporting such claims remains limited. In our previous experimental study, we investigated whether the application of PCs prior to repetitive glove occlusion could prevent the occlusion‐induced increase in skin susceptibility to a standardized sodium lauryl sulphate (SLS) challenge [13]. That study demonstrated that pre‐occlusion application of PCs did not attenuate the occlusion effect. Importantly, several formulations even resulted in a significantly enhanced irritant response compared with occlusion alone, indicating a potential negative impact on skin barrier function under occlusive conditions.

These findings raise critical questions regarding the mechanisms underlying the occlusion effect and, in particular, the importance of timing of topical skin product application in relation to glove occlusion. Therefore, the present study builds directly upon our previous work and aims to elucidate whether application of PCs after glove occlusion can mitigate the occlusion effect. By extending earlier findings, this study seeks to refine evidence‐based recommendations for the safe and effective use of skin protective products in occupational wet‐work settings.

## 2 Materials and Methods

### 2.1 Characterization of Participants and Experimental Procedure

All investigations were conducted in accordance with the ethical principles for medical research outlined in the Declaration of Helsinki and were approved by the local ethics committee of the Medical Faculty, Ruhr University of Bochum (No.: 22‐7664). Written informed consent was obtained from all participants prior to inclusion. All procedures and potential side effects were explained in detail.

Participants were examined at the Institute for Prevention and Occupational Medicine (IPA), Ruhr University Bochum, Germany, between February 2023 and November 2024.

#### 2.1.1 Participants

A total of 68 healthy volunteers aged between 18 and 65 years with clinically healthy skin on the forearms and hands were included. Exclusion criteria comprised any ongoing skin disease, known allergy to ingredients of the tested creams, pregnancy or breastfeeding, a history of cancer or cancer treatment within the preceding year, and intensive ultraviolet radiation exposure within the last 4 weeks.

Participants were additionally excluded if they had used topical corticosteroids or other topical anti‐inflammatory preparations on the arms or hands or had taken systemic immunosuppressive medication within 3 weeks prior to study initiation.

### 2.2 Protective Skin Creams

All protective creams (PCs) were previously investigated using the same experimental model, but applied before glove occlusion, to evaluate their efficacy [13]. In the present study, the same formulations were used but applied after occlusion. As new production batches were employed, minor variations in formulation cannot be completely excluded. In comparison, the ingredients remained the same as in our previous study.

Cream C (standardized model protective cream) was developed for the evaluation of skin protection efficacy prior to product labelling, and its composition has been described previously [14, 15, 16]. The galenic development was performed by the Department of Pharmaceutical Engineering (Prof. Kutz), University of Applied Sciences and Arts Ostwestfalen‐Lippe, Detmold, Germany.

#### 2.2.1 Cream A (Cream); Corresponds to Cream B From the Previous Study [13]

Aqua, paraffinum liquidum, behenyl alcohol, glycerine, aluminium chlorohydrate, ethylhexyl palmitate, *Simmondsia chinensis* oil, ceteth‐10, steareth‐20, dimethicone.

#### 2.2.2 Cream B (Lotion); Corresponds to Cream D From the Previous Study [13]

Aqua, glyceryl stearate SE, cetearyl ethylhexanoate, glycerine, cetearyl isononanoate, talc, kaolin, polyvinyl alcohol, cetearyl alcohol, panthenol, hydroxyethylcellulose, *Hamamelis virginiana* water, tocopheryl acetate, *Hamamelis virginiana* extract, stearic acid, SLS, sodium cetearyl sulphate, gallic acid, butylene glycol, phenoxyethanol, methylparaben, ethylparaben, propylparaben, butylparaben.

#### 2.2.3 Cream C (Standardized Model Protective Cream)

Aqua, kaolin, paraffinum liquidum, paraffin, cetearyl alcohol, cetyl alcohol, petrolatum, zinc oxide, sodium hexametaphosphate, SLS, sodium cetearyl sulphate, propylparaben, methylparaben.

### 2.3 Study Design

This experimental trial was single‐blind, controlled, and randomized. Participants were randomized using computer‐generated random numbers (Microsoft Excel RAND()), allocating values ≥ 0.5 to the right arm and values < 0.5 to the left arm. To evaluate the efficacy of PCs applied after glove occlusion, we used a modified in vivo model of repetitive occlusion followed by SLS irritation, as described in our previous studies [10, 13].

Glove occlusion was simulated using 3 × 5 cm pieces of nitrile glove material (Dermatril P, KCL GmbH, Eichenzell, Germany), fixed at the borders with Fixomull Stretch (BSN Medical GmbH, Hamburg, Germany).

#### 2.3.1 Phase 1 (Days 1–7)

##### 2.3.1.1 Application of Protective Creams After Glove Occlusion

During Phase 1, glove occlusion and protective cream (PC) application afterwards (dose: 3 mg/cm^2^) were performed for seven consecutive days (Figure 1). Applications were initially carried out by study personnel and subsequently by the participants. Participants received training to ensure correct dosing (using pre‐marked syringes) and were provided with individualized application protocols according to group allocation (see Supplement S1).

**FIGURE 1:**
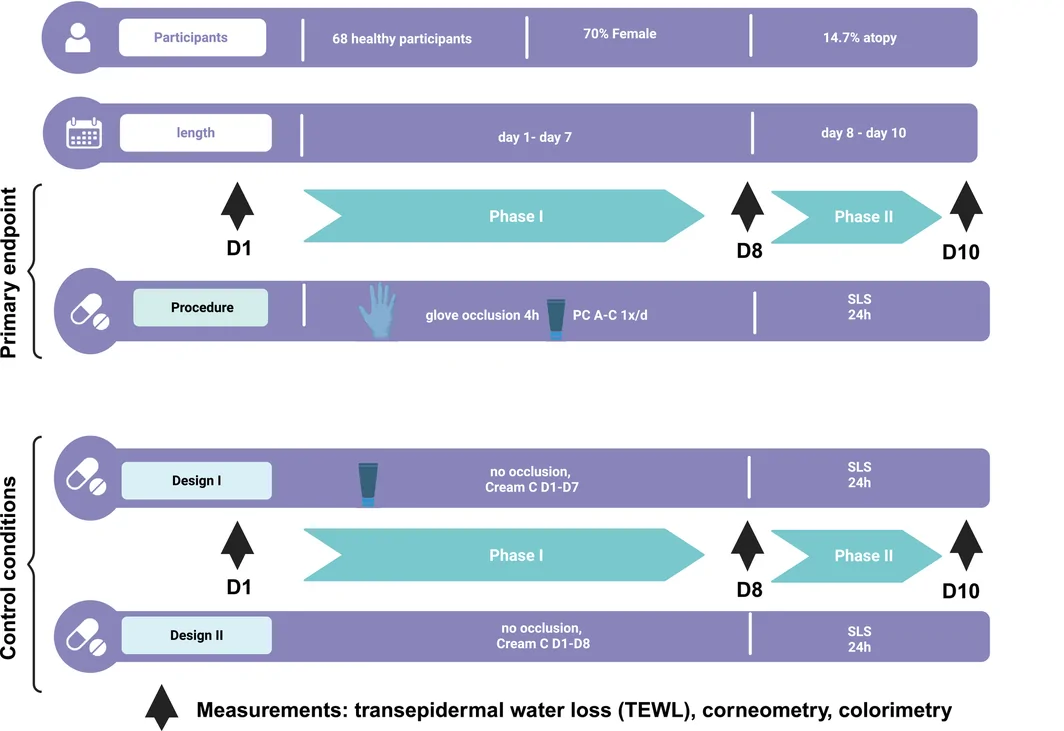
Schematic overview of the study design and experimental procedures. This figure illustrates the overall study design, including both experimental arms (primary endpoint and control conditions with Design I and Design II), and the timeline. Skin barrier function and irritation were evaluated at defined time points (Days 1, 8 and 10) using transepidermal water loss (TEWL), corneometry, and colourimetry. The study phases and procedural timeline are depicted to facilitate comparison between conditions and endpoints. The primary experimental condition consisted of repeated glove occlusion over multiple days with concurrent PC application, followed by SLS challenge to assess the ‘occlusion effect’. In Design I, cream C was applied without occlusion for 7 days, followed by a 24‐h sodium lauryl sulphate (SLS) challenge. In Design II, cream C was applied without occlusion for 8 days prior to SLS exposure. These conditions served as non‐occluded controls. Figure was made using Biorender software.

On each study day, marked areas on both forearms were occluded with nitrile glove material for 4 h to simulate occupational glove use. Immediately after removal of the occlusion material, PCs A and B, as well as control cream C, were applied.

Corresponding anatomical sites on the contralateral forearm served as control areas (occlusion for 1 week without PC application) (see Supplement S1). To avoid anatomical bias, identical regions (distal, distal‐middle, middle, proximal‐middle, proximal) were used on both forearms. Treatment allocation was permuted in a standardized manner (four permutation groups) by a study nurse according to the study protocol. The forearms were selected as the test site due to their established reliability in experimental irritation models.

##### 2.3.1.2 Control Conditions Without Occlusion With Cream C: Influence of Application Timing on Skin Irritation

Additional experimental conditions were designed exclusively for cream C to evaluate its effect on skin barrier function in the absence of occlusion and to assess whether efficacy depends on application timing relative to irritation with SLS.

Two treatment conditions without glove occlusion were investigated (Figure 1):

Design I: Daily application of cream C until Day 7 (i.e., 24 h prior to SLS challenge).

Design II: Daily application of cream C until Day 8, with the final application performed 10 min prior to SLS challenge.

These conditions were compared with:

- Areas treated with cream C under occlusion (hereafter referred to as “positive C”), and.
- Areas exposed to SLS without prior treatment (referred to as ‘positive control’) (see Tables 2 and 3).

#### 2.3.2 Phase 2 (Days 8–10): SLS Challenge

On day 8, after completion of phase 1 and 24 h after the last treatment, the marked areas were irritated with SLS for 24 h (50 μL of 0.5% SLS, purity ≥ 99%; Sigma‐Aldrich, St. Louis, MO, USA). Large Finn Chambers (12 mm; SmartPractice, Phoenix, AZ, USA) mounted on Scanopor tape (Actavis Norway AS, Vennesla, Norway) were used and removed by the participants on day 9.

Evaluation followed the guidelines for SLS testing of the Standardization Group of the European Society of Contact Dermatitis (ESCD) [17] (see Figure 1).

### 2.4 Measurements and Clinical Scoring

Room temperature and relative humidity were recorded during all measurements in accordance with ESCD guidelines [17]. All assessments were conducted in air‐conditioned rooms (mean temperature 20.2°C, SD 0.36; mean relative humidity 46.3%, SD 12.60; Table 1). Skin temperature was measured using a DermaTemp DT 1001 RS thermometer (Exergen, Watertown, MA, USA) and averaged 29.9°C (SD 0.97).

**TABLE 1:** Study population.

Study population	Total
*N* (%)	68
Sex (*N* (%))	
Male	20 (29.4)
Female	48 (70.6)
Age in years (median (IQR))	40.5 (23.0–53.5)
Age in years (range)	18–65
Atopic score (median, (IQR))	6.0 (4.0–9.0)
< 8 (*N* (%))	45 (66.2)
8–9 (*N* (%))	12 (17.7)
≥ 10 (*N* (%))	10 (14.7)
*Missing*	*1 (1.5)*
**Study conditions**	
Skin temperature in °C D1 (mean (STD))	29.9 (0.97)
Room temperature in °C D1 (mean (STD))	20.2 (0.36)
Humidity D1 (mean (STD))	46.3 (12.60)
Skin temperature in °C D10 (mean (STD))	29.9 (1.00)
Room temperature in °C D10 (mean (STD))	20.1 (0.47)
Humidity D10 (mean (STD))	42.9 (10.61)

*Note:* Characterization of the study population on baseline (D1) including atopy status, and study conditions on baseline (D1) and end of the study (D10).

Abbreviations: IQR, inter quartile range; STD, standard deviation.

Participants rested in a relaxed position for 20 min before measurements. All assessments were performed by the same investigator according to standardized guidelines at baseline (Day 1), on day 8 (before SLS challenge), and on Day 10 (Table 1).

#### 2.4.1 Bioengineering Methods

All measurements were conducted twice at each test site, and mean values were calculated for statistical evaluation. Skin surface temperature was assessed prior to transepidermal water loss (TEWL) measurements using a Derma Temp DT 1001 RS device (measurement range 18°C–43°C; Exergen, Watertown, MA, USA) (Table 1).

TEWL, serving as a marker of epidermal barrier function, was determined with an MPA9 system equipped with a Tewameter TM 300 (Courage & Khazaka, Cologne, Germany) in accordance with established guidelines.

Stratum corneum hydration was evaluated by corneometry, which assesses skin capacitance, using a Corneometer CM 825 connected to an MDD4 unit (Courage & Khazaka, Cologne, Germany). Measurements were performed over a surface area of 49 mm^2^ at a frequency of 0.9–1.2 MHz with an accuracy of ±3%, and results were expressed in arbitrary units (range 0–99).

Skin surface colour was analysed according to the Commission Internationale de l’Éclairage (CIE) tristimulus colour system. Cutaneous erythema was quantified by reflectance spectrophotometry using a Chromameter CR 300 (Konica Minolta Photo Imaging Europe, Langenhagen, Germany) [18]. Redness, reflecting haemoglobin content and inflammatory activity, was reported as the a* colour coordinate. Due to technical limitations, erythema measurements were available for a subset of participants (*n* = 42). All measurements were conducted according to established guidelines [17, 19, 20].

#### 2.4.2 Visual Examination (Clinical Score)

Clinical evaluation was performed on Day 10. The degree of skin irritation was assessed according to the guidelines of the European Society of Contact Dermatitis (ESCD) [21, 22] for acute SLS‐induced irritant reactions. Scores were defined as follows:

0 = no reaction;

1 = smooth surface with slight dryness or roughness;

2 = weak erythema with slight dryness, roughness, or scaling;

3 = erythema with increased roughness, scaling, oedema, or fissures;

4 = pronounced erythema with marked oedema, extensive scaling, and possibly vesicles, bullae, pustules, or crusts.

#### 2.4.3 Atopic Skin Diathesis

Atopic skin diathesis (AD) was assessed using the Erlanger atopy score [23, 24]. Serological testing (total IgE, specific IgE or prick testing) was not performed. AD was defined as present with a score of > 10 and considered doubtful with scores between 8 and 9.

### 2.5 Statistical Analysis

The primary hypothesis was that 1 week of treatment with protective cream would mitigate the occlusion effect observed in the SLS irritation model. Differences between treated areas (PC application plus occlusion) and control areas (occlusion only) represent dependent observations, as measurements were obtained from both forearms of each participant.

Sample size calculation was based on our previous study with similar hypotheses using G*Power 3.1 [25], assuming a power of 80% to detect statistically significant differences between treatments using a one‐sided Wilcoxon signed‐rank test for matched pairs (α = 0.05). Non‐parametric analyses were applied, as baseline data were predominantly non‐normally distributed (Shapiro–Wilk test, 10% significance level).

The one‐sided Wilcoxon signed‐rank test for matched pairs was used to compare intraindividual differences between treated and control areas after 7 days of treatment and/or occlusion (ΔD8–D1) and after SLS challenge (ΔD10–D1). For TEWL and erythema, we tested whether treated areas exhibited smaller differences compared with controls; for corneometry, greater differences in treated areas were tested. To account for multiple comparisons, we used the Benjamini and Yekutieli method to control the false discovery rate and present adjusted *p*‐values [26].

Descriptive data are presented as medians with first and third quartiles. Results are illustrated using box plots, with boxes representing interquartile ranges, horizontal lines indicating medians, whiskers extending to the 5th and 95th percentiles, and outliers plotted as individual points. Statistical analyses were conducted using SAS version 9.4 (SAS Institute Inc., Cary, NC) and R version 4.5.0 (https://www.r‐project.org/). Graphs were generated with GraphPad Prism version 10.6.1 (GraphPad Software Inc., La Jolla, CA).

## 3 Results

### 3.1 Subject Characteristics

A total of 68 healthy volunteers, predominantly Caucasian (only two volunteers had Fritzpatrick's phototype equal or above IV) were included in the study. The median age was 40.5 years (interquartile range [IQR] 23.0–53.5), with a predominance of female participants (70.6%) (Table 1).

Based on the atopy score (AS), 66.2% of participants showed no atopic diathesis (AS < 8), while 14.7% fulfilled the criteria for atopic diathesis (AS ≥ 10). One participant had missing AS data.

### 3.2 Phase 1—Occlusion and Protective Cream Application Over 7 Days

Intraindividual differences in skin barrier function assessed by bioengineering methods (transepidermal water loss [TEWL], erythema by colourimetry a*, and corneometry) as well as clinical scoring were analysed between treated and corresponding control sites (identical anatomical regions) from baseline (D1) to Day 8 (D8) (Table 2). No significant differences in TEWL were observed between areas treated with protective creams and untreated control areas subjected to occlusion alone. Similarly, no differences were detected for erythema (colourimetry a*). In contrast, differences in corneometry values were significantly greater in areas treated with creams A and B compared with their respective control areas (Table 2), indicating a moisturizing effect of these formulations under occlusive conditions.

**TABLE 2:** Differences between Day 8 and baseline (after 7 days of treatment with protective cream and occlusion or occlusion only). Median and IQR (interquartile range), *N* = 68.

	Cream and measurement	Treated region (median (IQR))	Control region (median (IQR))	*p*‐value^a^	*p*‐value adjusted with Benjamini‐Yekutieli
**Primary endpoint: Application of protective creams after glove occlusion**					
**Glove occlusion**	**Cream A**				
	Δ TEWL (D8–D1)	0.38 (−0.05–1.36)	0.29 (0.02–0.90)	0.6533	1.0000
	Δ Erythema (D8–D1)	−0.10 (−1.02–0.70)	−0.25 (−0.50–0.56)	0.5148	1.0000
	Δ Corneometry (D8–D1)	3.0 (−2.0–8.0)	0.0 (−4.0–6.0)	**0. 0009**	**0. 0406**
	**Cream B**				
	Δ TEWL (D8–D1)	0.22 (−0.16–1.10)	0.24 (−0.11–0.69)	0.7784	1.0000
	Δ Erythema (D8–D1)	−0.15 (−0.97–0.25)	0.10 (−1.03–0.85)	0.3471	1.0000
	Δ Corneometry(D8–D1)	7.0 (1.0–12.0)	1.0 (−2.5–6.0)	**< 0.0001**	**0. 0001**
	**Cream C**		**Control C**		
	Δ TEWL (D8–D1)	0.43 (−0.00–1.28)	0.28 (−0.05–0.95)	0.9324	1.0000
	Δ Erythema (D8–D1)	−0.34 (−0.71–0.34)	−0.13 (−0.71–0.69)	0.2730	1.0000
	Δ Corneometry (D8–D1)	1.5 (−4.5–6.0)	1.0 (−5.5–5.0)	0.2243	1.0000
**Control conditions including only cream C**					
**No occlusion**	**Design (I) Cream C**		**Compared to (a) Control C (b) Positive control (SLS)**		
	Δ TEWL (D8–D1)	0.22 (−0.01–1.22)	(a) 0.28 (−0.05–0.95) (b) 0.11 (−0.18–0.69)	0.6972 0.9734	1.0000 1.0000
	Δ Erythema (D8–D1)	−0.02 (−0.56–0.52)	(a) −0.13 (−0.71–0.69) (b) −0.10 (−0.74–0.86)	0.6540 0.2929	1.0000 1.0000
	Δ Corneometry (D8–D1)	−3.0 (−7.0–2.0)	(a) 1.0 (−5.5–5.0) (b) −2.00 (−7.00–2.50)	0.9985 0.6558	1.0000 1.0000
	**Design (II) Cream C 10′ before SLS**				
	Δ TEWL (D8–D1)	0.16 (−0.18–0.77)	(a) 0.28 (−0.05–0.95) (b) 0.11 (−0.18–0.69)	0.3581 0.8080	1.0000 1.0000
	Δ Erythema (D8–D1)	0.02 (−0.69–0.76)	(a) −0.13 (−0.71–0.69) (b) −0.10 (−0.74–0.86)	0.6448 0.2678	1.0000 1.0000
	Δ Corneometry (D8–D1)	−2.0 (−6.0–1.0)	(a) 1.0 (−5.5–5.0) (b) −2.00 (−7.00–2.50)	0.9984 0.7258	1.0000 1.0000
**Control conditions**					
	**Positive control (SLS)**				
	Δ TEWL (D10–D1)	0.11 (−0.18–0.69)	—	—	—
	Δ Erythema (D10–D1)	−0.10 (−0.74–0.86)	—	—	—
	Δ Corneometry (D10–D1)	−2.00 (−7.00–2.50)	—	—	—
	**Negative control**				
	Δ TEWL (D10–D1)	0.08 (−0.11–0.28)	—	—	—
	Δ Erythema (D10–D1)	−0.14 (−0.95–0.52)	—	—	—
	Δ Corneometry (D10–D1)	−2.50 (−8.00–3.00)	—	—	—

*Note: p*‐values < 0.05 are indicated in bold. Median values; first and second quartiles are in parentheses. Δ TEWL D8–D1 (g/m^2^/h): TEWL Day 8—TEWL baseline Day 1. Δ Erythema D8–D1: a* values: a*Day 8—a*baseline Day 1. Δ Corneometry D8–D1 (arbitrary units): corneometry Day 8—corneometry baseline Day 1.

^a^ *p*‐values from one‐sided Wilcoxon signed rank tests. Hypothesis for Δ TEWL (D8–D1) and Δ Erythema (D8–D1): protective cream and occlusion < control region, and for Δ Corneometry (D8–D1): protective cream and occlusion > control region.

### 3.3 Phase 2—SLS Challenge: Differences Between Day 10 and Baseline

#### 3.3.1 Primary Endpoint: Protective Cream Application After Occlusion Versus Occlusion Alone

Application of protective creams after occlusion resulted in a tendency but not significant reduction of SLS‐induced TEWL increase only for cream A compared with its respective occlusion‐only control (median ΔTEWL (D10–D1): 10.88 vs. 12.13; *p*‐value = 0.78, one‐sided Wilcoxon signed‐rank test) (see primary endpoint in Table 3, Figure 2A).

**TABLE 3:** Differences between Day 10 and baseline (after phase 2‐SLS irritation of the previously treated areas and control areas). Median and IQR (interquartile range).

	Cream and measurement	Treated region (median [IQR])	Control region (median [IQR])	*p*‐value^a^	*p*‐value adjusted with Benjamini‐Yekutieli
**Primary endpoint: Application of protective creams after glove occlusion**					
**Glove occlusion**	**Cream A**				
	Δ TEWL (D10‐D1)	10.88 (7.67–16.71)	12.13 (8.48–21.13)	**0. 0214**	0.7779
	Δ Erythema (D10‐D1)	1.81 (0.92–4.20)	2.29 (1.10–4.17)	0.2918	1.0000
	Δ Corneometry (D10‐D1)	−5.0 (−9.0–4.0)	−3.0 (−10.0–4.0)	0.2348	1.0000
	**Cream B**				
	Δ TEWL (D10‐D1)	13.44 (8.16–22.56)	12.42 (6.35–19.10)	0.9848	1.0000
	Δ Erythema (D10‐D1)	2.44 (2.51–3.59)	2.54 (1.18–4.08)	0.5283	1.0000
	Δ Corneometry (D10‐D1)	−2.0 (−8.5–5.0)	−2.5 (−9.0–4.5)	0.5038	1.0000
	**Cream C**		**Control C**		
	Δ TEWL (D10‐D1)	11.99 (7.14–19.83)	12.38 (7.00–19.42)	0.4453	1.0000
	Δ Erythema (D10‐D1)	2.60 (1.35–3.79)	2.47 (1.16–3.34)	0.8622	1.0000
	Δ Corneometry (D10‐D1)	−5.5 (−11.0–1.5)	−4.0 (−11.5–4.5)	0.9817	1.0000
**Control conditions including only cream C**					
**No occlusion**	**Design (I) Cream C**		**Compared to (a) Control C (b) Positive control (SLS)**		
	Δ TEWL (D10‐D1)	11.92 (5.93–19.48)	(a) 12.38 (7.00–19.42) (b) 10.23 (6.30–16.37)	0.1530 0.6668	1.0000 1.0000
	Δ Erythema (D10‐D1)	2.20 (0.90–3.86)	(a) 2.47 (1.16–3.34) (b) 2.31 (1.08–3.52)	0.7591 0.5099	1.0000 1.0000
	Δ Corneometry (D10‐D1)	−6.0 (−12–0.5)	(a) −4.0 (−11.5–4.5) (b) −3.0 (−10.0–3.0)	0.8265 0.8989	1.0000 1.0000
	**Design (II) Cream C 10′ before SLS**				
	Δ TEWL (D10‐D1)	7.09 (5.04–10.26)	(a) 12.38 (7.00–19.42) (b) 10.23 (6.30–16.37)	**< 0.0001** **< 0.0001**	**0. 0001** **0. 0054**
	Δ Erythema (D10‐D1)	1.64 (0.88–2.75)	(a) 2.47 (1.16–3.34) (b) 2.31 (1.08–3.52)	0.0879 **0. 0493**	1.0000 1.0000
	Δ Corneometry (D10‐D1)	−13.0 (−19.0–−6.0)	(a) −4.0 (−11.5–4.5) (b) −3.0 (−10.0–3.0)	1.0000 1.0000	1.0000 1.0000
**Control conditions**					
	**Positive control (SLS)**				
	Δ TEWL (D10‐D1)	10.23 (6.30–16.37)	—	—	—
	Δ Erythema (D10‐D1)	2.31 (1.08–3.52)	—	—	—
	Δ Corneometry (D10‐D1)	−3.0 (−10.0–3.0)	—	—	—
	**Negative control**				
	Δ TEWL (D10‐D1)	0.15 (−0.12–0.52)	—	—	—
	Δ Erythema (D10‐D1)	−0.19 (−0.76–0.39)	—	—	—
	Δ Corneometry (D10‐D1)	−3.0 (−9.5–1.0)	—	—	—

*Note: p*‐values < 0.05 are indicated in bold. Table showing median values; first and second quartile are in parentheses. The SLS Irritation on Day 8 was performed 24 h after the last wet exposure (water and occlusion). Δ TEWL D10–D1 (g/m^2^/h): TEWL Day 10‐TEWL baseline Day 1. Δ Erythema D10–D1: a* values on Day 10—baseline Day 1. Δ Corneometry D10–D1: corneometry Day 10—corneometry baseline Day 1.

^a^ *p*‐values from one‐sided Wilcoxon signed rank tests. Hypothesis for Δ TEWL (D10–D1) and Δ erythema (D10–D1): protective cream and occlusion < control region, and for Δ corneometry (D10–D1): protective cream and occlusion > control region.

**FIGURE 2:**
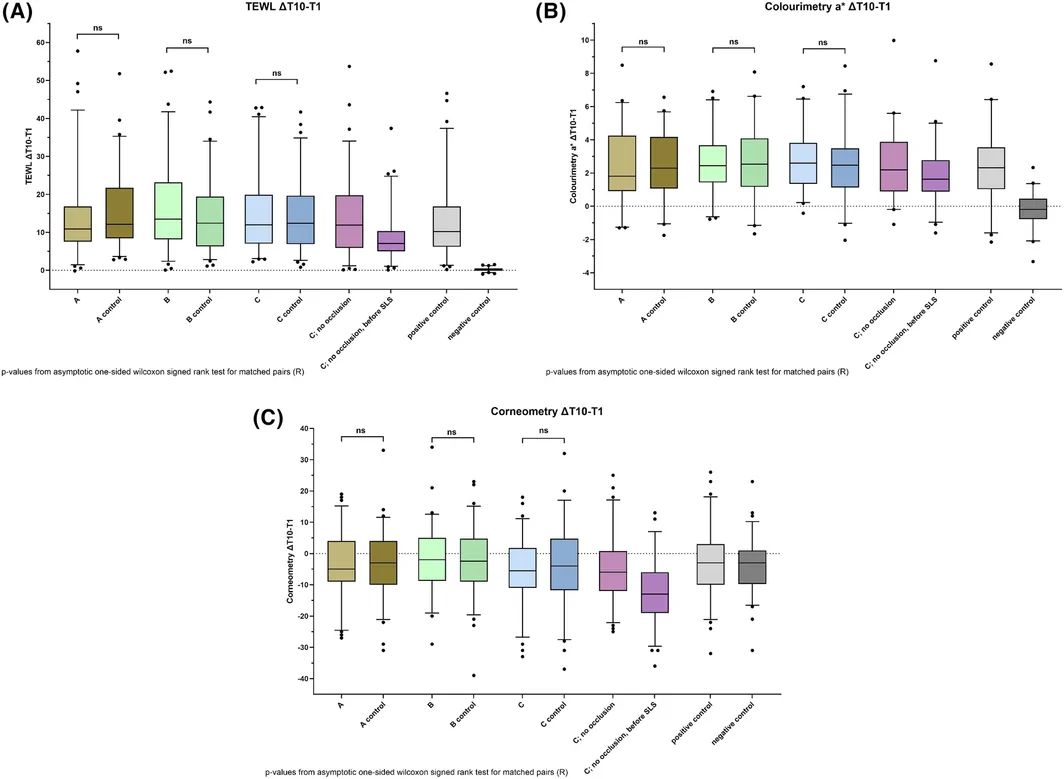
(A) Differences D10–D1 for transepidermal water loss (TEWL). Graph showing boxplots of TEWL differences between D10 and D1 for the creams, their corresponding controls (occlusion only), and the control conditions for cream C (see Figure 1 and Table 3) depicted as boxplots and whiskers represent the 5th–95th percentiles. Adjusted *p*‐values from one‐sided Wilcoxon signed‐rank tests for group differences in dependent samples. (B) Differences D10–D1 for colourimetry (value a*). Boxplots showing differences in values for erythema (value a*) between D10 and D1 for creams and their corresponding controls (occlusion only), and the control conditions for cream C (see Figure 1 and Table 3) with median and whiskers with 5–95 percentile. Adjusted *p‐*values from one‐sided Wilcoxon signed‐rank tests for group differences in dependent samples. (C) Differences D10–D1 for corneometry values. Corneometry—differences between D10 and D1 (recorded as arbitrary units, AU, 0–99) for creams and their corresponding controls (occlusion only), and the control conditions for cream C (see Figure 1 and Table 3) depicted as boxplots and whiskers with 5–95 percentile. Adjusted *p‐*values from one‐sided Wilcoxon signed‐rank tests for group differences in dependent samples.

For cream B, boxplot showed an opposing trend to our initial hypothesis (Figure 2A).

Regarding skin hydration, cream C showed the strongest difference to the respective control sites, but not in the hypothesized direction (Table 3, Figure 2C). There were no significant differences for erythema (see primary endpoint in Table 3, Figure 1B).

Clinical scoring demonstrated a tendency toward higher irritation scores in occluded areas compared with non‐occluded areas (Figure 3).

**FIGURE 3:**
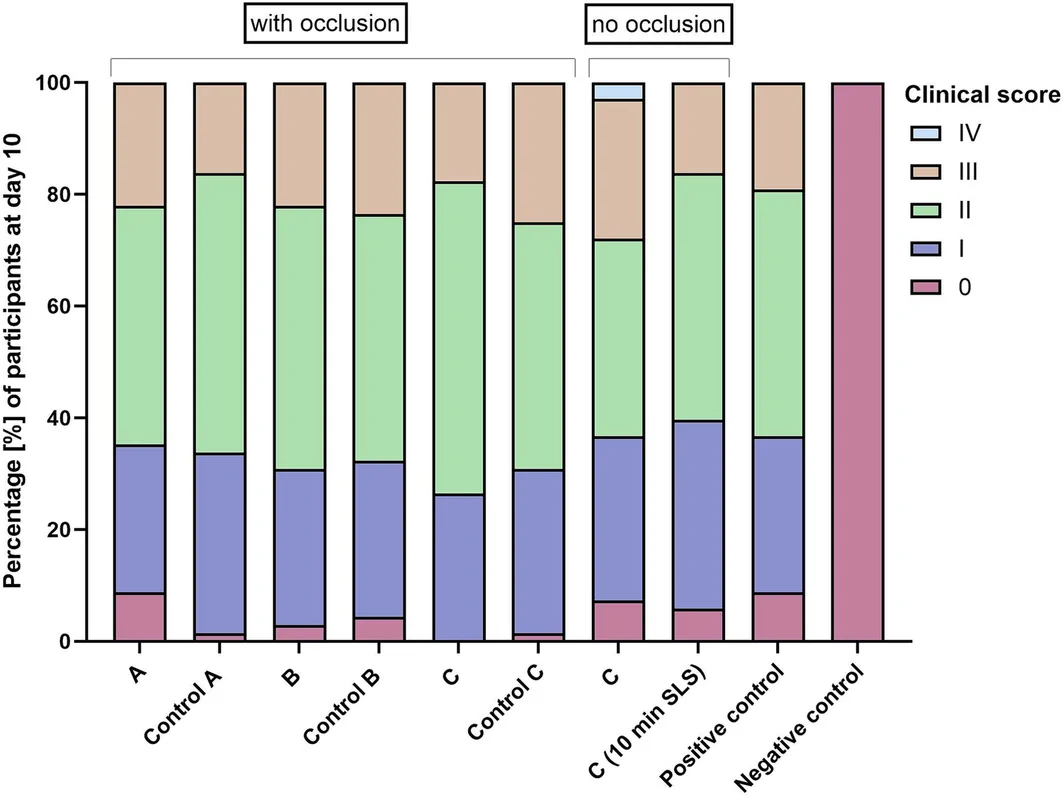
Clinical scores on Day 10 shown as relative percentages on the Scale 0–IV.

#### 3.3.2 Control Conditions: Cream C Application Without Occlusion Versus Control With Occlusion and SLS

In this part of the study, only cream C was tested without occlusion and applied over either (I) 7 or (II) 8 days (see Sections 2.3.1.2, Figure 1, and Supporting Information). The outcomes in these areas were compared with the primary endpoint results obtained for cream C. Specifically, comparisons were made with (a) untreated control areas subjected to occlusion only, and (b) non‐occluded areas challenged with SLS (positive control) (Table 3). Unlike the main experiment, control areas were not anatomically matched on the forearms.

For Design I, no significant differences were observed between cream C–treated areas and corresponding controls in TEWL, erythema or corneometry (see control conditions in Table 3).

In Design II, application of cream C up to 10 min before SLS challenge significantly reduced TEWL compared with both conditions for cream C with occlusion (median ΔTEWL: 7.09 vs. 12.38) in control C and 10.23 in SLS control (positive control) (Table 3, Figure 2A). A reduction in erythema was also observed compared with the SLS control (1.64 vs. 2.31); this finding was statistically not significant after *p*‐value adjustment. However, this treatment regimen was associated with a decrease in skin hydration, contrary to our initial hypothesis, as reflected by corneometry values (median Δ corneometry: −13.0 vs. −3.0 in SLS control and −4.0 in control C).

## 4 Discussion

Our earlier randomized controlled study demonstrated that application of PCs prior to glove occlusion generally failed to mitigate the occlusion effect and, in some cases, significantly increased SLS‐induced skin irritation [13]. No formulation showed a protective effect against occlusion in the previous investigation.

The present study confirms and extends these observations by directly contrasting PC application after occlusion with our previous findings on pre‐occlusion application. Building on our 2023 study, the current results provide further evidence that application of PCs after glove occlusion shows no or only limited protective effects against the occlusion‐induced increase in skin susceptibility, and in some cases even aggravated barrier impairment.

Overall, post‐occlusion application of the protective creams resulted in heterogeneous effects on skin barrier function, with trends largely resembling those observed following pre‐occlusion application. Application of cream A (in the previous study did not modify the occlusion effect) after occlusion led to a statistically non‐significant reduction in SLS‐induced TEWL compared with occlusion alone, suggesting a possibility of partial stabilization of the epidermal barrier after removal of occlusive stress.

In contrast, cream B (in our earlier study, which enhanced the occlusion effect) again resulted in barrier deterioration, whereas cream C (the control cream in the previous study; showing no effect in Part I and an increased occlusion effect in Part II) did not alter the occlusion response in the present setting.

These observed effects may be specific to the formulation.

At present, it remains unclear which specific properties a PC must possess to exert a protective effect under occlusive conditions. These findings highlight that post‐occlusion application is not universally protective and probably remains formulation‐dependent. It underscores the importance of galenic composition, emulsifier systems and potential penetration‐enhancing ingredients, and emphasize the need for independent efficacy testing. They also illustrate that manufacturer‐provided information regarding ‘use under gloves’ or ‘protection against occlusion’ is often limited and may be misleading.

According to previous experimental studies, the direct impact of glove occlusion on epidermal barrier function appears to be limited, with measurable impairment typically observed only under conditions of prolonged experimental occlusion (as reviewed by Tiedemann et al. [8] and Fartasch et al. [9]). With prolonged occlusion (> 24 h), increased TEWL may persist even in the absence of apparent skin dryness [7]. However, these changes are largely transient, as TEWL generally returns to baseline within a few hours after removal of occlusion [27]. Despite their transient nature, these alterations may facilitate the binding and penetration of irritants such as SLS, thereby contributing to the enhanced irritant response observed after occlusion. In line with previous findings [10], repetitive occlusion over 1 week did not significantly alter baseline skin barrier function in our study, as reflected by measurements on Day 8. Nevertheless, an increased susceptibility to irritation was observed, as demonstrated by an enhanced response to SLS challenge. Consistent with these findings, and despite the absence of significant differences in bioengineering parameters at Day 8 compared to baseline, an occlusion effect was evident in our study too. This was reflected by higher TEWL values in the cream C‐treated areas under occlusion compared with (i) cream C without occlusion, (ii) occluded control areas and (iii) the positive control (no additional statistical testing was performed for these comparisons). Together, these results support the concept that moderate occlusion induces a subclinical increase in skin susceptibility, which becomes apparent only after a subsequent irritant challenge.

The mechanisms underlying this ‘occlusion effect’ are not fully understood but are thought to involve (i) subclinical inflammatory processes and (ii) increased penetration of irritants due to structural alterations of the stratum corneum (SC) [10]. In addition, water dynamics likely play a central role in this process. TEWL, which reflects water diffusion across the SC, exhibits a non‐linear increase with rising hydration levels [28]. Water accumulation within the SC can induce structural changes in corneocytes, disrupt intercellular lipid organization and promote the formation of intercellular water pools [29]. Although prolonged occlusion (up to 72 h) has not been shown to significantly alter ceramide profiles or ceramide‐to‐cholesterol ratios in healthy skin [30], there is substantial evidence that lipid organization itself is affected [31]. Specifically, increased hydration shifts lipid packing from a highly ordered orthorhombic phase—associated with optimal barrier function—toward less ordered hexagonal or liquid‐crystalline phases [32]. Accordingly, skin with elevated TEWL exhibits a reduced proportion of orthorhombic lipid packing compared to healthy skin.

Interestingly, cream C in design II showed a protective effect (Figure 1, Table 3) and was accompanied by a significant reduction in skin hydration. This finding points to complex interactions between cream components, stratum corneum water dynamics and irritant penetration.

Given the experimental design, our results must be interpreted with caution. The model does not directly reflect typical occupational settings but rather represents a simplified scenario of prolonged occlusion combined with limited or absent intermittent irritant exposure. In real‐world workplaces, glove occlusion is usually interspersed with repeated exposure to irritants such as soaps and disinfectants, creating a more complex exposure pattern.

Despite these limitations, the overall balance of evidence suggests that the use of PCs in occupational settings may be beneficial [12]. Indeed, a Cochrane review concluded that the use of PCs in the workplace may help prevent irritant contact dermatitis [33].

Our findings support the protective effect of PCs against detergent‐induced irritation but highlight the critical importance of application timing. Additional control experiments using cream C without occlusion demonstrated that repeated application up to 24 h prior to SLS challenge resulted in irritation responses comparable to untreated SLS‐exposed areas. In contrast, application shortly before exposure (within 10 min) significantly reduced TEWL and erythema. This suggests that PCs exert their protective effect primarily when applied in close temporal proximity to irritant exposure, likely through the formation of a transient protective layer or by modifying interactions between the skin surface and the irritant.

These findings may have practical implications and could help explain previous results. For example, Held et al. reported that the use of a moisturizer under occlusion reduced irritation following detergent exposure [34]. In that study, the moisturizer was applied immediately before and after short‐term SLS exposure, followed by glove occlusion, suggesting that direct interaction between the moisturizer and the irritant plays a key role. Similarly, Antonov et al. demonstrated a protective effect of PCs when irritant exposure occurred between periods of glove occlusion [35]. These observations suggest that, although PCs do not mitigate the occlusion effect itself, their use in situations involving intermittent irritant exposure—particularly when applied immediately before such exposure—may outweigh the potentially adverse effects associated with occlusion.

Furthermore, in situations where the combined use of PCs and liquid‐proof gloves is unavoidable, post‐occlusion application may be preferable to pre‐occlusion use. This approach may reduce potential interactions between PCs and glove materials [12] that could compromise glove integrity and protective function.

Key strengths of this study include its highly controlled experimental design, intraindividual comparisons and sufficient statistical power to detect clinically relevant medium‐to‐large effects.

However, several limitations should be considered. First, the study was conducted under experimental conditions in a population consisting predominantly of healthy Caucasian volunteers, which may limit generalizability. In addition, the standardized exposure conditions and application protocols may not fully reflect real‐world workplace practices. We did not expose the backs of the hands, but rather the forearms. However, forearms are an accepted test site in experimental irritation studies and are recommended in the guidelines of the European Society of Contact Dermatitis [21].

The applied amount of skin protection cream (PC) (3 mg/cm^2^) [15] exceeds typical usage in occupational settings, where approximately 1 mg/cm^2^ is applied on average [36]. However, the use of a higher dose was intended to ensure optimal conditions for detecting a potential protective effect. The absence of such an effect under these conditions suggests that a protective benefit at lower, more realistic application amounts is unlikely. Moreover, the PCs were applied by the participants themselves, so we cannot rule out compliance issues that were not reported.

Furthermore, the study was partially conducted during the summer period. Although participants were instructed to avoid UV exposure of the forearms, increased ultraviolet exposure cannot be entirely excluded as a confounding factor, for example, through modulation of local immune responses. However, only a subset of participants was examined during this period, which likely limited its overall impact on the results. Finally, our findings are limited to the specific products tested. Although similar effects may be expected for comparable formulations, variability between products cannot be excluded. This underlines the importance of independent, standardized testing and a critical evaluation of manufacturer claims.

Taken together, the present findings refine the understanding of occlusion effects and their modulation by topical products. From an occupational dermatology perspective, the results indicate that the use of PCs specifically to counteract the occlusion effect cannot be generally recommended, regardless of whether application occurs before or after occlusion. Most tested formulations showed no beneficial effect and, in some cases, even increased occlusion‐induced skin susceptibility.

Nevertheless, in situations where the combined use of PCs and liquid‐proof gloves is unavoidable, post‐occlusion application may be preferable to pre‐occlusion use [12]. Moreover, although PCs do not mitigate the occlusion effect itself, their use in settings with intermittent irritant exposure—particularly when applied immediately before such exposure—may still provide a net protective benefit and outweigh the potentially adverse effects associated with occlusion.

## 5 Conclusions

This study demonstrates that the effectiveness of PCs in the context of glove occlusion is limited. Application of PCs either before or after occlusion generally does not prevent the occlusion effect. Only one formulation showed tendencies of protective effects; however, did not reach statistical significance.

In contrast, short‐term application immediately before irritant exposure—without preceding occlusion—may provide transient protection, highlighting the critical importance of timing. Overall, these findings challenge current occupational skin protection practices and manufacturer claims regarding PC use in combination with liquid‐proof gloves. Evidence‐based recommendations should emphasize cautious, timing‐specific use of PCs and the need for independent efficacy testing of manufacturer claims.

## Author Contributions

**Katharina Wichert:** data curation, formal analysis, software, validation, visualization, writing – review and editing. **Michal Gina:** conceptualization, methodology, investigation, data curation, project administration, validation, formal analysis, supervision, funding acquisition, visualization, resources, writing – review and editing, writing – original draft. **Julia Krabbe:** project administration, resources, funding acquisition, supervision, writing – review and editing. **Thomas Brüning:** writing – review and editing. **Thomas Behrens:** writing – review and editing.

## Conflicts of Interest

M.G., K.W., T.Be., T.B., J.K. declare no conflicts of interest. M.G., K.W., T.Be., T.B., J.K., as staff of the Institute for Prevention and Occupational Medicine (IPA), are employed at the German Social Accident Insurance. The IPA is an independent research institute of the Ruhr University Bochum. As scientists and authors, they are independent of the German Social Accident Insurance regarding study design, access to the collected data, responsibility for data analysis and interpretation, and with regard to the right to publish. The views expressed in this paper are those of the authors and not necessarily those of the financial supporters.

## Supporting information


**Supplement. S1** Example depicting the used areas on forearms, and how the procedures were carried out during Phase 1 (Day 1–7) and Phase 2 (Day 8–10). In total, four groups were formed, each with a different permutation of procedures in order to limit the impact of anatomical localisation. To test the primary endpoint, a patch test with glove occlusion was applied over 4 h followed by application of a protective cream on the left forearm (distal, medial distal, medial), whereas on the right forearm, only occlusion was applied to the corresponding locations. Medial proximal and proximal were used for control conditions.

## Data Availability

The data that support the findings of this study are available on request from the corresponding author. The data are not publicly available due to privacy or ethical restrictions.
