# Supplementary material for: Post‐Occlusion Application of Skin Protective Creams Does Not Affect Occlusion‐Induced Barrier Changes: A Randomized Controlled Experimental Study
Source: Contact Dermatitis. 2026 Jul 15;95(5):437–49. doi: 10.1111/cod.70220 (PMC13648313; doi:10.1111/cod.70220)
Supplement: Supplementary file 1 [file COD-95-437-s001.docx]

***Supplement***

*Supplement 1. Example depicting the used areas on forearms, and how the procedures were carried out during phase 1 (day 1-7) and phase 2 (day 8-10). In total four goups were formed, each with different permutation of procedures in order to limit the impact of anatomical localisation. To test primary endpoint, p*atch test with glove occlusion was applied over 4 hours followed by application of a protective cream on the left forearm (distal, medial distal, medial), whereas on the right forearm only occlusion was applied to the corresponding locations. Medial proximal and proximal were used for control conditions.

|  | **Anatomical site** | **LEFT ARM** | **RIGHT ARM  (control areas)** |
| --- | --- | --- | --- |
| **Occlusion 4h** | **Distal** | **Days 1–7:** **CREAM A**  **Day 8:** SLS for 24h | **Days 1–7:** occlusion only  **Day 8:** SLS for 24h |
|  | **Medial distal** | **Days 1–7: CREAM B** **Day 8:** SLS for 24h | **Days 1–7:** occlusion only **Day 8:** SLS for 24h |
|  | **Medial** | Positive C   **Days 1–7:** **CREAM C**.  **Day 8:** SLS for 24h | Control C   **Days 1–7:** occlusion only **Day 8:** SLS for 24h |
|  |  | **Control conditions** |  |
| **no occlusion** | **Design II)**  **Medial proximal** | **Days 1–7:** **CREAM C**  **Day 8:** Cream C, after 10 min SLS for 24h | **Days 1–7: no cream** **Day 8:** SLS for 24h |
|  | **Design I)**  **Proximal** | **Days 1–7:** **CREAM C**  **Day 8:** SLS for 24h | **Days 1–10:** no cream **Untreated control** |
